# Observations on Abscess of the Antrum Maxillary

**Published:** 1841-12

**Authors:** S. P. Hullihen

**Affiliations:** Wheeling, Va.


### ARTICLE III.

### *Observations on Abscess of the Antrum Maxillary.* By S. P. HULLIHEN, Wheeling, Va.

THE Antrum Maxillare is subject to a variety of diseases, the most common of which is the abscess of this cavity.

Mr. Bell, in speaking of this disease considers it to be nothing more than an altered secretion of the membrane, and observes :—"The term abscess as applied to the disease in question, has given rise to very mistaken notions of its nature, and not less erroneous principles of treatment. A reference to the structure of the antrum, would appear to be sufficient to point out the improbability, to say the least, of the occurrence of abscess in such a situation. That a mucous membrane covering in a thin layer, the whole internal surface of such a cavity, should become the seat of all the consecutive steps of true abscess, is a statement bearing on the face of it an obvious absurdity. The disease is in fact nothing more than an altered secretion of the membrane.

This opinion of Mr. Bell, is surely as erroneous as any that has ever been advanced upon the subject. A mere glance at the origin of the disease will satisfy, I think, the most superficial observer, that it is in the strictest sense of the word, abscess, and must be considered as perfectly indentical with alveolar abscess, proceeding from the same cause, the progress and development of both being the same.

The abscess of the antrum and of the alveolus both originate in the formation of pus in the internal cavity of a tooth. Whenever the nerve of a tooth becomes nearly exposed by caries, fracture, or other causes, it is subjected to much irritation and consequent inflammation, which sooner or later runs into excess, and suppuration of the nerve and vessels is the consequence. When matter is thus formed, if it cannot escape through an opening in the crown of the tooth it will immediately commence oozing out at the foramen of the fang, and effuse itself around and between the point of the root, and the bottom of the alveolar cell. The matter now is rapidly secreted, and soon finds its way through the thinner plate of the alveolus which is generally the external, and here it is again effused between the gum and alveolus, forming what has been very generally termed, "alveolar abscess."

Now if the fangs of such a tooth as just described, proximate the antrum so near, that the floor of this cavity is thinner than either plate of the alveolus, the matter always seeking that direction where there is the least resistance, finds its way into the antrum, and is effused between the bone and its lining membrane. Or if the fangs of such a tooth penetrate the antrum, the membrane of this cavity being always continuous over the fangs, the pus is effused between the membrane and the bone, thus forming in either case a true abscess in the antrum. The abscess now enlarges by secretion from the surrounding parts, until the distended membrane bursts, and the matter escapes from the antrum through the duct into the nose.

To show further the identity of abscess in the antrum and that of the alveoli, at the commencement of an alveolar abscess, a tooth in which the nerve and vessels are suppurating always becomes very painful and sore, appears a little loose and longer than the rest, giving rise to the generally received opinion that alveolar abscess originates in an inflammation of the periosteum of the fang or of the alveolar cell. At this period of the disease pains dart along the courses of the nerves, to the ear, temple and side of the head. Within twenty-four hours the face generally becomes swollen, and the character of the pain changes to one of a constant throbbing, in the gum over the sore tooth. The pain continues without much abatement for several days, until the abscess breaks and begins to discharge; then all pain immediately subsides.

The same kind of symptoms in every particular experienced in alveolar abscess, always accompany the formation of an abscess in the antrum. A tooth situated under or near this cavity first becomes very painful and sore, and appears loose and longer than the rest; then great pain is experienced in the ear, temple, and scalp; the cheek becomes more or less tumefied; and then the description of pain changes to one of a throbbing character, deeply seated in the jaw, and so continues for several days until the abscess breaks. A discharge through the nose instantly follows, and the pain in the jaw subsides.

Again, after an alveolar abscess occurs, an incurably diseased state of the periosteum, covering the point of the fang in which the abscess originated is frequently induced, and for several months after it is liable to become inflamed and painful on taking cold. When such inflammation takes place, a pain not generally of much severity is experienced. The tooth again becomes sore, the gum suddenly inflames and puffs up where the original abscess broke through it. In a few hours a discharge of matter usually follows and the pain is entirely removed. Such formation of pus in the gum may occur in the same place several times in a month, and constitutes what has been termed by some gum-boils.

After an abscess has occurred in the antrum, it is subject to the same kind of symptoms and the very same results follow as after alveolar abscess. Upon taking cold, the patient will experience a dull throbbing pain in the jaw, just sufficiently severe to be annoying. A tooth situated under or near the antrum becomes sore, in a few hours an increased discharge of matter from the antrum through the nose takes place, and the pain in the jaw subsides. This increased discharge from the antrum may continue for a few days, and then gradually diminish until it is scarcely perceptible, and will so remain until pain in the jaw and soreness in the tooth is again experienced, and then an increased discharge from the antrum will as certainly follow.

These strong marked symptoms and discharges from the antrum, are only experienced during the first year or eighteen months after the formation of the abscess. After that period, the parts from the effects of disease, are rendered less liable to become inflamed and painful from any constitutional or local cause. The membrane of the antrum is likewise more involved in disease, rendering the discharge from this cavity of a more uniform quantity.

The foregoing facts together with many others I might adduce, force me to the conclusion that a true abscess of the antrum does frequently occur, and that it must be considered as perfectly identical with alveolar abscess; both originating in the same cause, the progress and development of both being the same; differing only in their locality and the consequences that naturally result therefrom.

### *Treatment of Abscess of the Antrum.*

From the situation and structure of the antrum, it is indispensably necessary whenever it becomes diseased to make an opening into its cavity. The size of the opening should always be made with a strict reference to the state of the disease.

*The first stage of Abscess.*—During the formation of an abscess, and when the patient is subject to pain in the jaw followed by an increased discharge, and the character of that discharge is healthy and well digested pus, a perforation into the antrum of such dimensions as to suffer the matter to escape freely, will be sufficiently large.

The method of opening the antrum in such cases, is after this manner. The tooth which from all the circumstances appears most probably to have caused the disease, should be extracted and a perforation large enough to admit a common quill, made into the antrum through that alveolar cell in which the fang most diseased was situated.

The trocar employed for this purpose, may be shaped like a common drill and used by grasping it firmly in the hand and carefully introducing it into the proper cell, then by pressing upwards and making a few rotatory motions the operation will be performed.

The after treatment consists in injecting into the antrum once or twice a day, mild washes of such a strength and character as the case may require; and in keeping the orifice made into the antrum, freely open as long as there is the least perceptible discharge. This may be accomplished by cutting the flesh out of the orifice, from time to time, instead of endeavouring to keep it open by the insertion of wooden plugs, and the like, which always prove a source of irritation, prevents the escape of matter as soon as formed, and as far as I have been able to observe, is never necessary to keep foreign substances out of the antrum.

*Second stage of Abscess.*—Where the disease is of long standing, or where the discharge is rather uniform in quantity and very fetid; and when the matter is so acrid as to excoriate the nostril and lips; it will be necessary to make a large opening into the antrum before a cure can be effected.

*Method of performing the operation.*—A tooth, and sometimes two should be extracted, and a perforation made through the alveolar cell as before described. After removing the gum as far as necessary, a small saw about the size of a common penknife blade may be introduced into the antrum through the opening made by the trocar, and a portion of the alveolus and floor of the antrum sawed out together, sufficiently large to admit the end of the finger into the sinus.

The after treatment is to some extent of the same character, as in the first stage of abscess. But when a pathological view is taken of a long diseased and spongy membrane, and the physiological action necessary for its restoration, something more appears to be necessary, at least to bring about a speedy cure. The first object that is to be attained in the treatment of such a membrane, is to promote absorption. The second of equal importance, is to regulate the action of the secreting vessels and to keep them unloaded, and as free as possible from tumefaction. Then changing the action of both by deobstruents or local alteratives, the membrane may be restored to a healthy state. Taking this view of the subject, I have been induced to use *friction*, and with decided success. This may be done by brushing the membrane once a day or more, with a small stiff brush made expressly for the purpose. By this means the secreting vessels are stimulated and throw off considerable, the blood vessels still more ; the membrane is reduced in thickness, the application of washes rendered more effective, and thus a cure may be accomplished much sooner than by the usual mode of treatment.

In the next number of the Journal, I will offer a few obervations on "muco-purulent secretion," of the antrum, and show the difference between this disease and abscess of this cavity.

